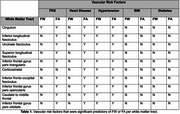# The Role of Cardiovascular Risk Factors in White Matter Tract Microstructure: A Multi‐Cohort Study in Older Adults

**DOI:** 10.1002/alz70856_107361

**Published:** 2026-01-09

**Authors:** James D LeFevre, Yukti Vyas, Aditi Sathe, Niranjana Shashikumar, Kimberly R. Pechman, Yisu Yang, Alaina Durant, Praitayini Kanakaraj, Michael E Kim, Chenyu Gao, Nancy R Newlin, Karthik Ramadass, Nazirah Mohd Khairi, Zhiyuan Li, Tianyuan Yao, Shannon L Risacher, Shannon L Risacher, Panpan Zhang, Kurt Schilling, Walter W. Kukull, Sarah Biber, Bennett A. Landman, Barbara B. Bendlin, Sterling C Johnson, Julie A Schneider, Lisa L. Barnes, David A. A. Bennett, Andrew J. Saykin, Michael L Cuccaro, Timothy J. Hohman, Angela L. Jefferson, Derek B. Archer, Alzheimer's Disease Neuroimaging Initiative (ADNI), The Alzheimer's Disease Sequencing Project (ADSP), The ADSP Phenotype Harmonization Consortium

**Affiliations:** ^1^ Vanderbilt Memory and Alzheimer's Center, Vanderbilt University School of Medicine, Nashville, TN, USA; ^2^ Department of Neurology, Vanderbilt University Medical Center, Nashville, TN, USA; ^3^ Department of Computer Science, Vanderbilt University, Nashville, TN, USA; ^4^ Department of Electrical and Computer Engineering, Vanderbilt University, Nashville, TN, USA; ^5^ Department of Radiology and Imaging Sciences, Indiana University School of Medicine, Indianapolis, IN, USA; ^6^ Indiana Alzheimer's Disease Research Center, Indiana University School of Medicine, Indianapolis, IN, USA; ^7^ Department of Biostatistics, Vanderbilt University Medical Center, Nashville, TN, USA; ^8^ Vanderbilt University Institute of Imaging Science, Vanderbilt University Medical Center, Nashville, TN, USA; ^9^ Department of Radiology and Radiological Sciences, Vanderbilt University Medical Center, Nashville, TN, USA; ^10^ National Alzheimer's Coordinating Center, University of Washington, Seattle, WA, USA; ^11^ Department of Biomedical Engineering, Vanderbilt University, Nashville, TN, USA; ^12^ Vanderbilt Brain Institute, Vanderbilt University Medical Center, Nashville, TN, USA; ^13^ Wisconsin Alzheimer's Disease Research Center, School of Medicine and Public Health, University of Wisconsin, Madison, WI, USA; ^14^ Wisconsin Alzheimer's Institute, School of Medicine and Public Health, University of Wisconsin, Madison, WI, USA; ^15^ Wisconsin Alzheimer's Disease Research Center, School of Medicine and Public Health, University of Wisconsin‐Madison, Madison, WI, USA; ^16^ Rush Alzheimer's Disease Center, Rush University Medical Center, Chicago, IL, USA; ^17^ Indiana Alzheimer's Disease Research Center, Indiana University School of Medicine, Indianapolis, IN, USA; ^18^ Dr. John T. MacDonald Foundation, Department of Human Genetics, University of Miami, Miami, FL, USA; ^19^ The John P. Hussman Institute for Human Genomics, University of Miami, Miami, FL, USA; ^20^ Vanderbilt Genetics Institute, Vanderbilt University Medical Center, Nashville, TN, USA; ^21^ Department of Medicine, Vanderbilt University Medical Center, Nashville, TN, USA

## Abstract

**Background:**

Vascular risk factors (VRF) exert deleterious effects on the vasculature throughout the body, including cerebrovasculature. These effects are characterized by structural and functional alterations in the cerebrovasculature, which in turn, may impact white matter (WM) microstructure. We sought to determine the relative contributions of VRF to alterations in WM tract microstructure in a large, multi‐study cohort using diffusion tensor imaging (DTI).

**Method:**

We collated datasets from the Vanderbilt Memory and Aging Project (VMAP) and several Alzheimer's Disease (AD) Sequencing Project‐Phenotype Harmonization Consortium (ADSP‐PHC) cohorts, including diffusion magnetic resonance imaging and VRF (*n* = 1,304, 72.3±9.5 years, 53% female). ADSP‐PHC cohorts included in this study were the AD Neuroimaging Initiative (ADNI), the National Alzheimer's Coordinating Center (NACC), the Religious Orders Study/Rush Memory and Aging Project/Minority Aging Research Study (ROS/MAP/MARS), and the Wisconsin Registry of Alzheimer's Prevention (WRAP). To assess the contributions of VRF to WM microstructure, we quantified the cross‐sectional association between VRF and white matter microstructure. Specifically, VRF were used to predict differences in free‐water (FW) and fractional anisotropy (FA_FWcorr_) across 10 AD‐relevant WM tracts. VRF variables studied included hypertension, diabetes, heart disease, body mass index (BMI), and Atherosclerotic Cardiovascular Disease Framingham Risk Score (FRS). Covariates included age, sex, education, race, *apolipoprotein E*‐ɛ4 status, and cognitive status. Models were corrected for multiple comparisons using the FDR approach.

**Result:**

Hypertension and heart disease were significantly associated with FA_FWcorr_ in all tracts, while FRS was a significant predictor for FA_FWcorr_ in 9/10 tracts (top associations shown in Figure 1). Diabetes and BMI were not significant predictors for FA_FWcor_ in any of the tracts. Hypertension was a significant predictor for FW in all tracts, and FRS was a significant predictor only in the cingulum. Diabetes, BMI, and heart disease were not significant predictors for FW in any of the tracts.

**Conclusion:**

This study demonstrates that VRF, especially hypertension, heart disease, and FRS, differentially impact WM tract microstructure, providing insight into their unique contributions to WM microstructure in a large multi‐cohort of older adults. These findings underscore the critical importance of managing vascular risk factors to preserve white matter integrity to potentially prevent cognitive decline.